# Decomposition and Spatio-temporal analysis of health care access challenges among reproductive age women in Ethiopia, 2005–2016

**DOI:** 10.1186/s12913-020-05639-y

**Published:** 2020-08-17

**Authors:** Getayeneh Antehunegn Tesema, Zemenu Tadesse Tessema, Koku Sisay Tamirat

**Affiliations:** Department of Epidemiology and Biostatistics, Institute of Public Health, College of Medicine and Health Sciences, University of Gondar, Gondar, Ethiopia

**Keywords:** Health care access challenge, Multivariate decomposition analysis, Spatio-temporal analysis, Ethiopia

## Abstract

**Background:**

The high maternal mortality, home delivery, unwanted pregnancies, incidence of unsafe abortion, and unmeet family planning needs are maternal health gaps attributed to health care access barriers and responsible for the observed health care disparities. Over the last decades remarkable achievements have made in relation to maternal health problems and the reduction of health care access barriers. Thus, this study aimed to assess the decomposition and spatial-temporal analysis of health care access challenges among reproductive-age women in Ethiopia.

**Methods:**

Secondary data analysis was conducted based on the three consecutive Ethiopian Demographic and Health Surveys (2005–2016 EDHSs). A total weighted sample of 46,235 reproductive-age women was included in this study. A logit based multivariate decomposition analysis was employed for identifying factors contributing to the overall decrease in health care access challenges over time. For the spatial analysis, ArcGIS version 10.6 and SaTScan™ version 9.6 were used to explore hotspot areas of health care access challenges in Ethiopia over time. Variables with *p*-value < 5% in the multivariable Logit based multivariate decomposition analysis were considered as significantly contributed predictors for the decrease in health care access challenges over time.

**Result:**

The mean age of the women was 27.8(±9.4) years in 2005, 27.7(±9.2) years in 2011, and 27.9 (±9.1) years in 2016. Health care access challenges have been significantly decreased from 96% in 2005 to 70% in 2016 with the Annual Rate of Reduction (ARR) of 2.7%. In the decomposition analysis, about 85.2% of the overall decrease in health care access challenge was due to the difference in coefficient and 14.8% were due to differences in the composition of the women (endowment) across the surveys. Socio-demographic characteristics (age, residence, level of education, female household head, better wealth and media exposure) and service utilization history before the survey (facility delivery and had ANC follow up) contribute to the observed decrease over time. The spatial analysis revealed that health care access challenges were significantly varied across the country over time. The SaTScan analysis identified significant hotspot areas of health care access challenges in the southern, eastern, and western parts of Ethiopia consistently over the surveys.

**Conclusion:**

Perceived health care access challenges have shown a remarkable decrease over time but there was variation in barriers to health care access across Ethiopia. Media exposure improved mothers’ health care access in Ethiopia. Public health programs targeting rural, uneducated, unemployed, and women whose husband had no education would be helpful to alleviate health care access problems in Ethiopia. Besides, improving mother’s media exposure plays a significant role to improve mothers’ health care access. Health care access challenges have significantly varied across the country. This suggests that further public health interventions are important for further reduction of health care access barriers through the uplifting socio-demographic and economic status of the population.

## Background

Remarkable progress has been made in the reduction of maternal and child mortality in the last two decades, given that preventable maternal death dropped from 386 per 100,000 live births in 1990 to 216 deaths in 2015 according to the global burden of disease study [1, 2]. However, maternal and child mortality is the unfinished agenda of the millennium development goal (MDG) is also the agenda under sustainable development goal (SDG) for further reduction of preventable maternal deaths below 70 per 100, 000 live births [3, 4]. The Sub-Saharan region is one of the highly affected with a death toll of 546 per 100, 000 live births in 2015 [2, 5].

Ethiopia is the country with a high magnitude of maternal deaths and achieved about 50% maternal mortality reduction with recent figures of 401 deaths per 100,000 live births [6]. Maternal health problems are still the leading public health concerns in developing countries. Health service availability, utilization, and accessibility contributed to maternal health problems. Health care accessibility is defined as the opportunity to have the health care needs fulfilled and measured in terms of utilization which depends on the affordability, physical accessibility, and acceptability of services and not merely adequacy of supply [7–9]. Access to comprehensive, quality health care services is important for promoting and maintaining health, preventing and managing the disease, reducing unnecessary disability and premature deaths, and achieving health equity for all women [10–13]. The high maternal mortality, home delivery, unwanted pregnancies, incidence of unsafe abortion, and unmeet family planning needs are maternal health gaps attributed to health care access barriers and responsible for the observed health care disparities. According to health care access barrier model, financial, structural, and cognitive are the three categories of health care access barrier (HCB) associated with decreased screening, late presentation to care, and lack of treatment, which in turn result in poor health outcomes and health disparities [7–11, 14]. Besides, geographical disparity, high costs of health care services, lack of transportation, and low socio-economic conditions of the population were barriers to health access problems [14–18].

A study conducted in South Africa, Cape Town only 35.2% of women accessed maternal health services. Another study in the same setting showed that availability, affordability, and acceptability of maternal health services were 30.5, 14, and 18.5%, respectively [9]. A comparative study in Nigeria and Ethiopia showed that maternal health services inequalities were observed between urban and rural, given that 48% women in urban and 55% in the rural area of Ethiopia perceived that institutional delivery is not necessary which is higher as compared to 42.7% women in urban and 45.9% of rural areas of Nigeria [19].

According to the 2016 Ethiopia Demographic and Health Survey, about 70% of women had health access problems which showed significant reductions from 96% in 2005 with geographical and socio-demographic variations [6, 20]. The Federal Democratic Republic of Ethiopia Government made interventions like urbanization, health facility expansion, providing maternal services free of charge, increasing health insurance enrollment, implementation of the health extension programs, women education and empowerment are some of the contributing factors for the reduction of health care access challenges among women [21–23]. However, there is scarce evidence about the spatial-temporal distributions and the contribution of each of the variables for the observed changes over time.

Therefore, this study aimed to assess the decomposition and spatial-temporal analysis of health care access challenges among reproductive-age women in Ethiopia based on EDHS 2005–2016. Findings from this study could help health system planners for evidence-based interventions and resource allocations. In addition, important lessons also are learned about the effect of characteristics and population structure changes for the reduction of health care access challenges.

**Fig. 1:**
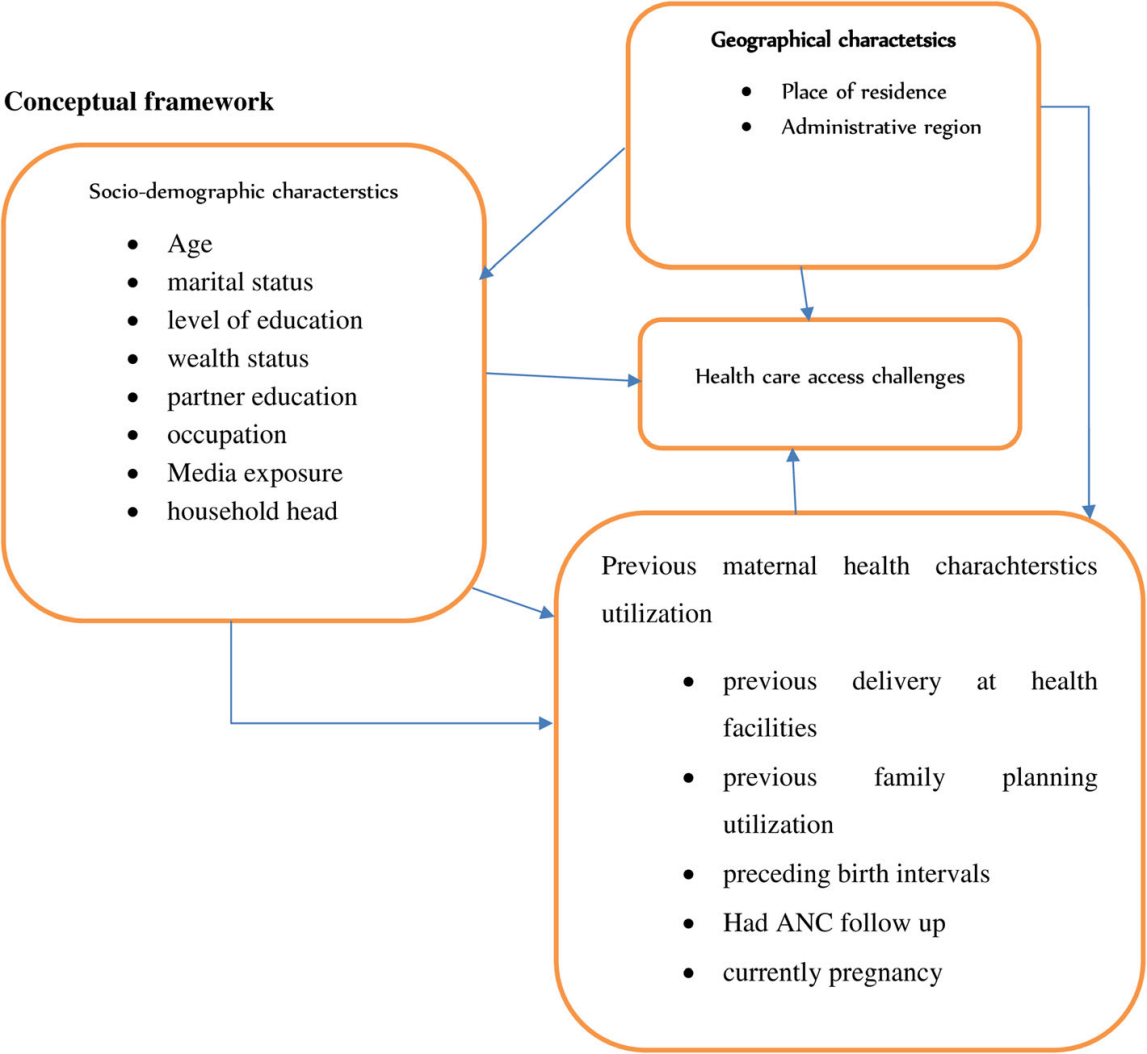
The conceptual framework adapting from literatures for analyzing factors contributing to health care access challenges

Evidnces from the previous litratures showed that socio-demographic attributes (age, marital status, level of education, occupation, wealth index, health insurance ceoverage, and media exposre and others), geographical chracterstics (residence and administarive regions) and previous maternal charactersics and service utilization (pregnancy during data collection. ANC follow up and facility delivery) were factors perceived to affect the healthcare acces among reproductive aage (Fig. 1).

## Methods

### Study design, setting and period

Secondary data analysis was conducted based on the three consecutive EDHSs data conducted in 2005, 2011, and 2016 [24–26]. These surveys are a nationally representative study conducted in Ethiopia, which is situated in the Horn of Africa. Ethiopia is the second most populous country in Africa next to Ngeria, and has 9 Regional states (Afar, Amhara, Benishangul-Gumuz, Gambela, Harari, Oromia, Somali, Southern Nations, Nationalities, and People’s Region (SNNP) and Tigray) and two Administrative Cities (Addis Ababa and Dire-Dawa). Ethiopia is an agrarian country and 84% of the population lives in a rural area, and 80% of the country’s total population lives in the regional states of Amhara, Oromia, and SNNP [27]. About 60% of the total population are living in the pastoral regions (Somali, Afar, Oromiya, Southern region, Gambella, and Benishangul-Gumuz regions) where people are sparsely populated and the community are least benefitted the health sector development [28]. Ethiopia is a multi-religious country with the domination of orthodox Christian and Muslim religious followers and having more than 80 ethnics groups that exercise their own culture and language. Ethiopia currently has one of the fastest growing economies in Africa and agriculture accounts for 40.5% of national GDP [29]. Ethiopia has registered an average annual growth rate of 11% of GDP, but 24% of the population still live below the national poverty line [30, 31]. Healthcare funding for the county is highly dependent on donors followed by households in the form of out-of-pocket care expenditure [32].

Ethiopia has 3 tiers health systems, Primary health care unit (Primary hospital, health center, health post, primary clinic, and medium clinic); Secondary health care (General hospital, specialty clinics, and specialty centers); and Tertiary health care (Specialized hospital). The number of hospitals varies from region to region in response to differences in population size [33].

### Sample and population

For this study, the data were obtained from eligible women aged 15–49 years who were participated in the survey. A stratified two-stage cluster sampling technique was employed for all three EDHS surveys using the population and housing census as a sampling frame. In total, 21 sampling strata have been created. In the first stage, a total of 540 Enumeration Areas (EAs) in EDHS 2005, 624 EAs in EDHS 2011, and 645 EAs in EDHS 2016 were selected with probability proportional to the EA size and with independent selection in each sampling stratum. At the second stage, on average 28–32 households were systematically selected. Based on this a total weighted sample of 14,062 reproductive-age women in EDHS 2005, 16,490 in EDHS 2011, and 15,863 in EDHS 2016 were included for the analysis. The detailed sampling procedure was presented in the full EDHS report [24–26]. For the spatial analysis, the geographic coordinate (longitude and latitude) data were taken from the selected enumeration areas. The EDHSs data set and the geographic coordinate data were accessed through an online request to the measure DHS program by explaining the objective of the study and we receive an authorization letter.

### Measurement of variables

The dependent variable was a score, health care access challenges were categorized dichotomously as Yes/No. To measure health care access challenges, each reproductive-age women were asked whether each of the following factors is a big problem in seeking medical advice or treatment for themselves when they are sick: 1) getting permission to go to the doctor, 2) getting money for advice or treatment, 3) distance to a health facility and 4) not wanting to go alone [34]. Then we created a composite variable that labeled as “health care access challenges” if the women responded to at least one the item as big problem classified as “had health care access challenges “and when women had responded as not a big problem to all of the questions then she was classified as “had no health access challenge” [35, 36]. Based on prior similar studies [37–39], the independent variables included in this study were maternal age (recoded as 15–24, 25–34, and 35–49), residence (recoded as urban, and rural), maternal education (recoded as no, primary education, and secondary and above), husband education (recoded as no, primary education, and secondary and above), marital status (recoded as never married, married/living together, and separated/widowed/divorced), wealth status (recoded as poor, middle and rich), visiting health facility in the last 12 months (recoded as Yes, and No), ANC visit (recoded as Yes and No), place of delivery (recoded as home and health facility), maternal occupation status (recoded as working and not working), contraceptive use and intention (recoded as using modern method, using traditional method, non-users and intends to use latter, and doesn’t intends to use), household head (recorded as male and female), preceding birth interval (recoded as < 2 years and ≥ 2 years), media exposure (generated by aggregating the three variables (reading news paper, listening to radio and watching television and recoded as No and Yes), and current pregnancy.

### Data collection procedure

This study was performed based on the three EDHSs data obtained from the official DHS measure system website www.measuredhs.com after permission was given via online request through specifying our analysis objective. We used the set of individual (IR) data and extracted the outcome and the independent variables. The location data (latitude and longitude) was obtained from the measure DHS program.

### Data management and analysis

The data were weighted using sampling weight, primary sampling unit, and strata before any statistical analysis to restore the representativeness of the survey and to tell the STATA to take into account the sampling design when calculating standard errors to get reliable statistical estimates. Cross tabulations and summary statistics were conducted to describe the study population. Descriptive and summary statistics were conducted using STATA version 14, ArcGIS version 10.6, SaTScan version 9.6, and R software.

### Decomposition analysis

Data from EDHS 2005, and 2016 were appended together with the decomposition analysis. The trend was assessed separately in three phases (phase 1 (2005–2011), phase 2 (2011–2016), and phase 3 (2005–2016)). A multivariate decomposition analysis of the decrease in health care access challenges over time was fitted to identify the significant factors contributing to the decrease in health care access challenges for the last 11 years (2005–2016). Logit based multivariate decomposition analysis technique for non-linear response model (MVDCMP) was used for identifying factors significantly contributing to the decrease in health care access challenges since it was a binary outcome. It was a regression analysis of the decrease in the health care access challenges between EDHS 2005 and 2016. The model utilizes the output from a logit based multivariate decomposition model to parcel out the observed decrease in the percentage of health care access problems across the survey into two components.

The multivariate decomposition analysis decomposes the overall decrease in health care access challenge overtime into the decrease due to the difference in women’s composition (endowment) across the surveys and the decrease due to the difference in the effect of the characteristics (coefficient) between the surveys. In the overall decomposition analysis, we can measure the percentage in an overall decrease in health care access challenges over time attributed to the compositional difference in women (difference in characteristics or endowment) and the percentage of overall decrease due to the difference in the effect of explanatory variables (difference in coefficient) between the surveys.

Hence, the observed decrease in health care access challenges between surveys is additively decomposed into a characteristics (or endowments) component and a coefficient (or effects of characteristics) component.

For logistic regression, the Logit or log-odd of health care access problem is taken as:

$$ \mathrm{Logit}\;\left(\mathrm{A}\right)\hbox{-} \mathrm{Logit}\;\left(\mathrm{B}\right)=\mathrm{F}\;\left(\mathrm{XA}\upbeta \mathrm{A}\right)\hbox{-} \mathrm{F}\;\left(\mathrm{XB}\upbeta \mathrm{B}\right). $$

$$ =\frac{\left[\mathrm{F}\;\left(\mathrm{XA}\upbeta \mathrm{A}\right)\hbox{-} \mathrm{F}\;\left(\mathrm{XA}\upbeta \mathrm{A}\right)\right]}{\mathrm{E}}+\frac{\left[\mathrm{F}\;\left(\mathrm{XB}\upbeta \mathrm{B}\right)\hbox{-} \mathrm{F}\;\right(\mathrm{XB}\upbeta \mathrm{B}\Big].}{\mathrm{C}} $$

The E component refers to the part of the differential owing to differences in endowments or characteristics. The C component refers to that part of the differential attributable to differences in coefficients or effects.

The equation can be presented as:

$$ \mathrm{Logit}\;\left(\mathrm{A}\right)\hbox{-} \mathrm{Logit}\;\left(\mathrm{B}\right)=\left[\upbeta 0\mathrm{A}\hbox{-} \upbeta 0\mathrm{B}\right]+\sum \mathrm{XijB}\ast \left[\upbeta \mathrm{ijA}\hbox{-} \upbeta \mathrm{ijB}\right]+\sum \upbeta \mathrm{ijB}\ast \left[\mathrm{XijA}\hbox{-} \mathrm{XijB}\right]. $$

- *Xij*B is the proportion of the jth category of the ith determinant in the DHS 2005,

- *Xij*A is the proportion of the jth category of the ith determinant in DHS 2016,

- *ΒijB* is the coefficient of the jth category of the ith determinant in DHS 2005,

- *ΒijA* is the coefficient of the jth category of the ith determinant in DHS 2016,

- *Β0B* is the intercept in the regression equation fitted to DHS 2005, and.

- *Β0A* is the intercept in the regression equation fitted to DHS 2016.

The recently developed multivariate decomposition for the non-linear model was used for the decomposition analysis of health care access challenges using the mvdcmp STATA command [40]. In this study variable with *p*-value <, 0.2 in the bivariable multivariate decomposition analysis were considered for the multivariable multivariate decomposition analysis. In the multivariable multivariate analysis variables with *p*-value< 5% in the endowment and coefficient component were considered as significant contributing factors for the decrease in health care access challenges over time. Variance Inflation Factor (VIF) and tolerance were done to check whether there is significant multicollinearity between the independent factors. The mean VIF in this study was less than 10 and tolerance greater than 0.1, it indicates there is no significant multicollinearity.

### Spatial analysis

ArcGIS version 10.6 software and SaTScan version 9.6 software were used to explore the Spatio-temporal distribution of health care access challenges. The global spatial autocorrelation (Global Moran’s I) was done to assess whether women’s health care access challenges were dispersed, clustered, or randomly distributed in the study area [25]. Global moran’s I is a spatial statistics used to measure spatial autocorrelation by taking the entire data set and produce a single output value which ranges from − 1 to + 1. Moran’s I value close to − 1 indicates that health care access challenges is dispersed, whereas moran’s I close to + 1 indicate health care access challenges are clustered and if moran’s I close to 0 revealed that health care access challenge is randomly distributed. A statistically significant Moran’s I (*p* < 0.05) showed that women’s health care access challenge is non-random.

Kriging interpolation was employed to explore the burdens of health care access challenges in the unsampled areas of the country based on the observed data. The spatial interpolation technique is used to predict women’s health care access challenges on the un-sampled areas in the country based on the value observed form sampled EAs. Therefore, part of a certain area can be predicted by using observed data using a method called interpolation. There are various deterministic and geostatistical interpolation methods. Among all of the methods, ordinary Kriging and empirical Bayesian Kriging are considered the best method since it incorporates the spatial autocorrelation and it statistically optimizes the weight [26]. In this study, the ordinary kriging spatial interpolation method was used for the predictions of women’s health care access challenge in unobserved areas of Ethiopia since it had the lowest residual.

Bernoulli based spatial scan statistical analysis was employed to detect the primary and secondary significant spatial clusters of health care access challenges using Kuldorff’s SaTScan version 9.6 software. The spatial scan statistic uses a circular scanning window that moves across the study area. A woman with health care access challenge was taken as cases and women with no health care access challenges were taken as controls to fit the Bernoulli model. The default maximum spatial cluster size of < 50% of the population was used since it allowed both small and large clusters to be detected and ignored clusters that contained more than the maximum limit. For each potential cluster, a likelihood ratio test statistic and the *p*-value were used to determine if the number of observed health care access challenge cases within the potential cluster was significantly higher than expected or not. The scanning window with maximum likelihood was the most likely performing cluster, and the *p*-value was assigned to each cluster using Monte Carlo hypothesis testing by comparing the rank of the maximum likelihood from the real data with the maximum likelihood from the random datasets. The primary and secondary clusters were identified and assigned *p*-values and ranked based on their likelihood ratio test, based on 999 Monte Carlo replications [27].

### Ethical approval and consent to participate

Since the study was a secondary data analysis of publically available survey data from the MEASURE DHS program, ethical approval and participant consent were not necessary for this particular study. We requested DHS Program and permission was granted to download and use the data for this study from http://www.dhsprogram.com. There are no names of individuals or household addresses in the data files. The geographic identifiers only go down to the regional level (where regions are typically very large geographical areas encompassing several states/provinces. In surveys that collect GIS coordinates in the field, the coordinates are only for the enumeration area (EA) as a whole, and not for individual households, and the measured coordinates are randomly displaced within a large geographic area so that specific enumeration areas cannot be identified.

## Results

### Characteristics of the study population

The mean age of the women was 27.8(±9.4) years in 2005, 27.7(±9.2) years in 2011, and 27.9 (±9.1) years in 2016. About one-third of the reproductive age women in all three surveys were found in the Oromia region. There was a slight increase in urban residence from 17.7% in 2005 to 22.2% in 2016. Regarding maternal education, the proportion of women who had formal education has decreased from 65.9% in 2005 to 47.8% in 2016 whereas mothers who had attained secondary and above education have increased from 11.9% in 2005 to 17.2% in 2016. The percentage of media exposure among reproductive-age women was increased by 46.3% in 2005 to 48.2% in 2016 (Table 1).

**Table 1:** Percentage distribution of characteristics of respondents in 2005, 2011 and 2016 Ethiopian Demographic and Health Surveys

Variables	EDHS 2005 (***n*** = 14,062)	EDHS 2011(***n*** = 16,490)	EDHS 2016(***n*** = 15,683)
	Weighted frequency (%)	Weighted frequency (%)	Weighted frequency (%)
**Region**			
Tigray	917 (6.5)	1102 (6.7)	1129 (7.2)
Afar	146 (1.0)	145 (0.9)	128 (0.8)
Amhara	3482 (24.8)	4422 (26.8)	3714 (23.7)
Oromia	5008 (35.6)	6005 (36.4)	5701 (36.4)
Somali	486 (3.5)	328 (2.0)	459 (2.9)
Benishangul	124 (0.9)	173 (1.1)	160 (1.0)
SNNPRs	2992 (21.3)	3232 (19.6)	3288 (21.0)
Gambela	44 (0.3)	69 (0.4)	44 (0.3)
Harari	39 (0.3)	48 (0.3)	38 (0.3)
Addis ababa	756 (5.4)	895 (5.4)	930 (5.9)
Dire dawa	69 (0.5)	69 (0.4)	90 (0.6)
**Residence**			
Rural	11,565 (82.3)	12,554 (76.1)	12,207 (77.8)
Urban	2497 (17.7)	3936 (23.9)	3476 (22.2)
**Religion**			
Orthodox	6915 (49.2)	7826 (47.5)	6786 (43.3)
Muslim	4008 (28.5)	4584 (27.8)	4893 (31.2)
Protestant	2652 (18.9)	3634 (22.0)	3674 (23.4)
Others	487 (3.4)	446 (2.7)	330 (2.1)
**Maternal age (in years)**			
15–24	5809 (41.3)	6935 (42.1)	6143 (39.2)
25–34	4321 (30.7)	5190 (31.5)	5302 (33.8)
35–49	3932 (28.0)	4365 (26.5)	4238 (27.0)
**Maternal education**			
No education	9269 (65.9)	8384 (50.8)	7498 (47.8)
Primary	3120 (22.2)	6265 (38.0)	5490 (35.0)
Secondary and above	1673 (11.9)	1841 (11.2)	2695 (17.2)
**Husband education**			
No education	6396 (45.5)	5947 (36.1)	4685 (29.9)
Primary	2725 (19.4)	4579 (27.8)	3772 (24.0)
Secondary and above	4941 (35.1)	5964 (36.2)	7226 (46.1)
**Marital status**			
Never married	3510 (25.0)	4465 (27.1)	4036 (25.7)
Married/living together	9064 (64.5)	10,271 (62.3)	10,223 (65.2)
Divorced/widowed/separated	1488 (10.5)	1754 (10.6)	1423 (9.1)
**Wealth status**			
Poor	5069 (36.1)	6019 (36.5)	5442 (34.7)
Middle	2731 (19.4)	3027 (18.4)	2978 (19.0)
Rich	6262 (44.5)	7444 (45.1)	7263 (46.3)
**Visiting health facility in the last 12 months**			
No	10,588 (75.3)	11,073 (67.2)	9157 (58.4)
Yes	3474 (24.7)	5417 (32.8)	6526 (41.6)
**Having ANC follow up**			
No	5224 (71.5)	4512 (57.2)	2818 (37.1)
Yes	2081 (28.5)	3383 (42.8)	4771 (62.9)
**Place of delivery**			
Home	6804 (93.1)	6943 (87.9)	5066 (66.8)
Health facility	501 (6.9)	952 (12.1)	2523 (33.2)
**Occupation status**			
Working	3980 (71.7)	6203 (37.6)	5220 (33.3)
Not working	10,082 (28.3)	10,287 (62.4)	10,463 (66.7)
**Contraceptive use and intention**			
Using modern method	1361 (9.7)	3077 (18.7)	3899 (24.9)
Using traditional method	92 (0.7)	146 (0.9)	75 (0.5)
No-user and intend to use latter	6772 (48.1)	8097 (49.1)	6470 (41.2)
Does not intend to use	5837 (41.5)	5170 (31.3)	5239 (33.4)
**Household head**			
Male	11,109 (79.0)	12,477 (75.7)	11,960 (76.3)
Female	2953 (21.0)	4013 (24.3)	3723 (23.7)
**Preceding birth interval (in years)**			
< 2	1660 (11.8)	1556 (9.4)	1698 (10.8)
≥ 2	12,402 (88.2)	14,934 (90.6)	13,985 (89.2)
**Currently pregnant**			
No	12,879 (91.6)	15,285 (92.7)	14,548 (92.8)
Yes	1183 (8.4)	1205 (7.3)	1135 (7.2)
**Media exposure**			
No	7558 (53.7)	5295 (32.1)	8127 (51.8)
Yes	6504 (46.3)	11,195 (67.9)	7556 (48.2)

### Trends of health care access challenges

**Fig. 2:**
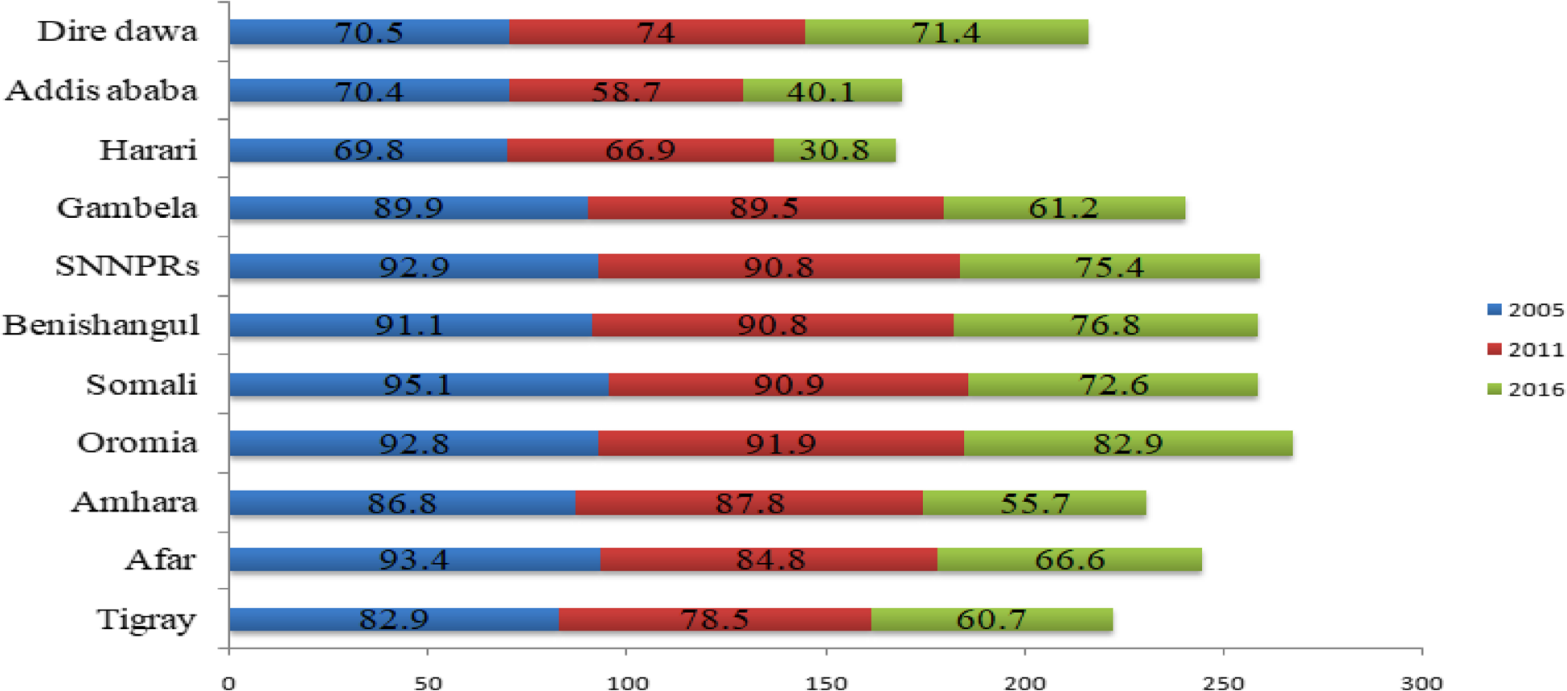
The trends of women health care access challenges across regions in Ethiopia

The overall health care access challenge among reproductive-age women has been decreased from 96% (95% CI: 95.2, 96.8) in 2005 to 70% (95% CI: 69.3, 70.7) in 2016 with Annual Rate of Reduction (ARR) of 2.7%. The trend in the health care access challenge has decreased in Addis Ababa, Harari, Amhara, Afar, Tigray, and Gambela regions over time (Fig. 2). About the place of residence, the percentage of health care challenges were decreased at a 24.1 point percentage among urban residents from 2005 to 2016. According to maternal education, there was a decline in health care access challenges among all education categories with the highest decrement in women with secondary and higher education at a 19.8% decrease in health care access problems in the entire study period (Table 2).

**Table 2:** Trends in health care access challeneges among reproductive age women by selected characteristics in 2005, 2011, and 2016 Ethiopia Demographic and Health Surveys

Variable	EDHS 2005	EDHS 2011	EDHS 2016	Point difference in health access problem		
				Phase 1 (2011–2005)	Phase 2 (2016–2011)	Phase 3 (2016–2005)
**Residence**						
Rural	93.6	92.9	77.0	−0.7	−15.9	−16.6
Urban	69.7	70.8	45.6	1.1	−25.3	−24.1
**Religion**						
Orthodox	85.9	83.5	59.0	−2.4	−24.5	− 26.9
Muslim	92.9	91.3	78.3	−1.6	− 13.0	− 14.6
Protestant	92.6	91.4	77.8	−1.2	−13.6	− 14.8
Others	90.6	92.7	86.8	2.1	−5.9	−3.8
**Women age (in years)**						
15–24	86.8	84.5	67.9	−2.1	−16.6	−18.7
25–34	89.8	88.8	69.8	−1.0	−19.0	−20.0
35–49	92.6	91.3	73.2	−1.3	−18.1	− 19.4
**Maternal education**						
No education	93.9	94.0	78.0	0.1	−16.0	− 15.9
Primary	88.7	86.4	71.2	−2.3	−15.2	−17.5
2^0^ and above	65.2	62.9	45.4	−2.3	−17.5	− 19.8
**Husband education**						
No education	94.3	93.9	77.5	−0.4	−16.4	− 16.8
Primary	92.9	91.2	74.3	−1.7	−16.9	− 18.6
Secondary and above	80.9	78.6	62.9	−2.7	−15.3	−18.0
**Marital status**						
Never married	83.0	81.7	64.2	−1.3	−17.5	− 18.8
Married/living together	91.2	89.9	71.9	−1.3	−18.0	− 19.3
Widowed/divorced/separated	93.3	89.3	73.0	−4.0	−16.3	−20.3
**Wealth status**						
Poor	95.0	93.8	84.1	−1.2	−9.7	−10.9
Middle	94.5	93.3	77.3	−1.2	−16.0	− 17.2
Rich	82.5	80.3	56.5	−2.2	−23.8	− 26.0
**Place of delivery**						
Home	93.7	94.0	81.4	0.3	−12.6	−12.3
Health facility	75.8	72.8	62.1	−3.0	−10.7	−13.7
**Having ANC follow up**						
No	95.1	94.7	83.5	−0.4	−11.2	− 11.6
Yes	86.0	87.1	69.9	1.1	−17.2	−16.1
**Contraceptive use and intention**						
Using modern method	83.1	85.3	64.6	2.2	−20.7	−18.5
Using traditional method	67.8	62.3	48.4	−5.5	−13.9	−19.4
Non-user and intends to use later	88.6	86.6	69.0	−2.0	−17.6	−19.6
Does not intend to use	92.0	91.3	75.6	−0.7	−15.7	− 16.4
**Visited health facility in the last 12 months**						
No	89.9	88.9	72.6	−1.0	− 16.3	− 17.3
Yes	87.7	85.1	66.3	−2.6	−18.8	−21.4
**Sex of household head**						
Male	89.9	88.2	70.8	−1.7	− 17.4	−19.1
Female	87.3	85.8	67.5	−1.5	−18.3	− 19.8
**Occupation status**						
Working	89.2	86.7	72.4	−2.5	−14.3	−16.8
Not working	89.4	88.2	65.3	−1.2	−22.9	−24.1
**Currently pregnant**						
Yes	93.7	90.3	69.8	−3.4	−20.5	− 23.9
No	89.0	87.4	73.0	−1.6	− 14.4	−16.0
**Media Exposure**						
No	94.0	93.6	77.0	−0.4	−16.6	− 17
Yes	77.0	84.8	50.7	7.8	−34.1	−26.3

### Decomposition analysis

The overall multivariate decomposition analysis revealed that about 85.2% of the overall decrease in health care access challenges among reproductive-age women was due to the difference in coefficient (difference in the effect of characteristics) across the surveys whereas the remaining 14.8% of the overall decrease in health care access challenge was due to the difference in composition of the respondent (endowment) across the surveys (Table 3). In the detailed decomposition analsyis, among the change due to composition (endowment); change in composition of rural residence *(B = -0.005, 95% CI: − 0.007, − 0.004),* women with secondary and higher education *(B = 0.005, 95% CI: 0.003, 0.006),* women aged 25–34 (*B = 0.001, 95% CI: 0.0004, 0.002),* history of health facility delivery *(B = − 0.017, 95% CI: − 0.026, − 0.008),* women whose husband attained primary education *(B = 0.002, 95% CI: 0.0005, 0.003),* had history of ANC follow up *(B = − 0.017, 95% CI: − 0.026, − 0.008),* female household head *(B = 0.002, 95% CI: 0.0002, 0.004*), middle wealth status *(B = 0.002, 95% CI: 0.001, 0.004),* rich wealth status *(B = 0.006, 95% CI: 0.005, 0.008)* and having media exposure *(B = 0.002, 95% CI: 0.0009, 0.003)* were signifcatly contributed for the decrease in health care access challenges over 11 years (from 2005 to 2016). Among the overall decrease in health care access challenges attributed to the difference in coefficients; the difference in effects of rural residence *(B = 0.007, 95% CI: 0.001, 0.01),* women aged 25–34 *(B = 0.02, 95% CI: 0.003, 0.03),* women having an occupation (B = − 0.009, 95% CI: − 0.02, − 0.003), and had a history of ANC follow-up *(B = 0.01, 95% CI: 0.002, 0.02)* were the factors significantly contributed for the decrease in health care access problem (Table 4).

**Table 3:** The overall decomposition analysis result of the decrease in health care access challenges over the last 11 years (2005–2016)

Health care access challenges	Coef (95% CI)	Pct.
**E**	−0.03 (− 0.04, − 0.02)	14.8
**C**	− 0.17 (− 0.19, − 0.15)	85.2
**R**	−0.20 (− 0.21, − 0.19)	

*C Coefficient, CI Confidence Interval, E Endowment, Pct Percentage, R:Residual*

**Table 4:** Detailed decomposition analysis of health care access challenges among reproductive age women in Ethiopia, 2005–2016

Health care access challenges		Difference due to characteristics(E)		Difference due to coefficient (C)	
		Coef.	Pct.	Coef.	Pct.
Residence	Rural	−0.005 (− 0.007, − 0.004)^**^	− 2.7	0.007 (0.001, 0.01)^*^	− 3.6
	Urban	0		0	
Maternal education	No education	0		0	
	Primary education	−0.002 (−0.005, 0.002)	0.8	0.0007 (−0.005, 0.006)	−0.4
	Secondary and higher	0.005 (0.003, 0.006)^*^	−2.5	0.002 (−0.001, 0.006)	−1.2
Maternal age (in years)	15–24	0		0	
	25–34	0.001 (0.0004, 0.002)^**^	−0.6	0.02 (0.003, 0.03)^*^	−7.9
	≥ 35	−0.0001 (− 0.0004, 0.0002)	0.1	− 0.0009 (− 0.009, 0.008)	0.4
Place of delivery	Home	0		0	
	Health facility	−0.017 (− 0.026, − 0.008)^*^	8.6	−0.001 (− 0.006, 0.003)	−5.5
Husband education	No education	0		0	
	Primary	0.002 (0.0005, 0.003)^*^	−0.8	0.003 (− 0.005, 0.01)	−1.7
	Secondary and above	0.0018 (−0.002, 0.005)	−0.9	0.004 (− 0.002, 0.01)	−2.1
Occupation status	Working	−0.0001 (− 0.002, 0.001)	0.01	− 0.009 (− 0.02, − 0.003)^*^	4.7
	Not working	0		0	
Currently pregnant	No	0		0	
	Yes	−0.0003 (−0.0002, 0.0002)	0.02	−0.0001 (− 0.005, 0.005)	0.06
Having ANC follow up	No	0		0	
	Yes	−0.017 (−0.026, − 0.008)^**^	8.6	0.01 (0.002, 0.02)^*^	−5.5
Household head	Male	0		0	
	Female	0.002 (0.0002, 0.004)^**^	−1.14	−0.004 (−0.009, 0.0006)	2.1
Wealth status	Poor	0		0	
	Middle	0.002 (0.001, 0.004)^*^	−1.2	−0.002 (−0.009, 0.006)	0.8
	Rich	0.006 (0.005, 0.008)*	−3.1	−0.01 (− 0.03, 0.004)	5.3
Media exposure	No	0		0	
	Yes	0.002 (0.0009, 0.003)^*^	−1.0	−0.002 (−0.01, 0.01)	0.9
Preceding birth interval	< 2 year	0		0	
	≥ 2 year	−0.0002 (− 0.0007, 0.0003)	0.12	0.008 (− 0.02,0.04)	−3.8
total			14.8		85.2

* is if *p*-value < 0.05 and ** if *p*-value < 0.01

### Variations in rural-urban inequality in health care access challenges across regions over time in Ethiopia

**Fig. 3:**
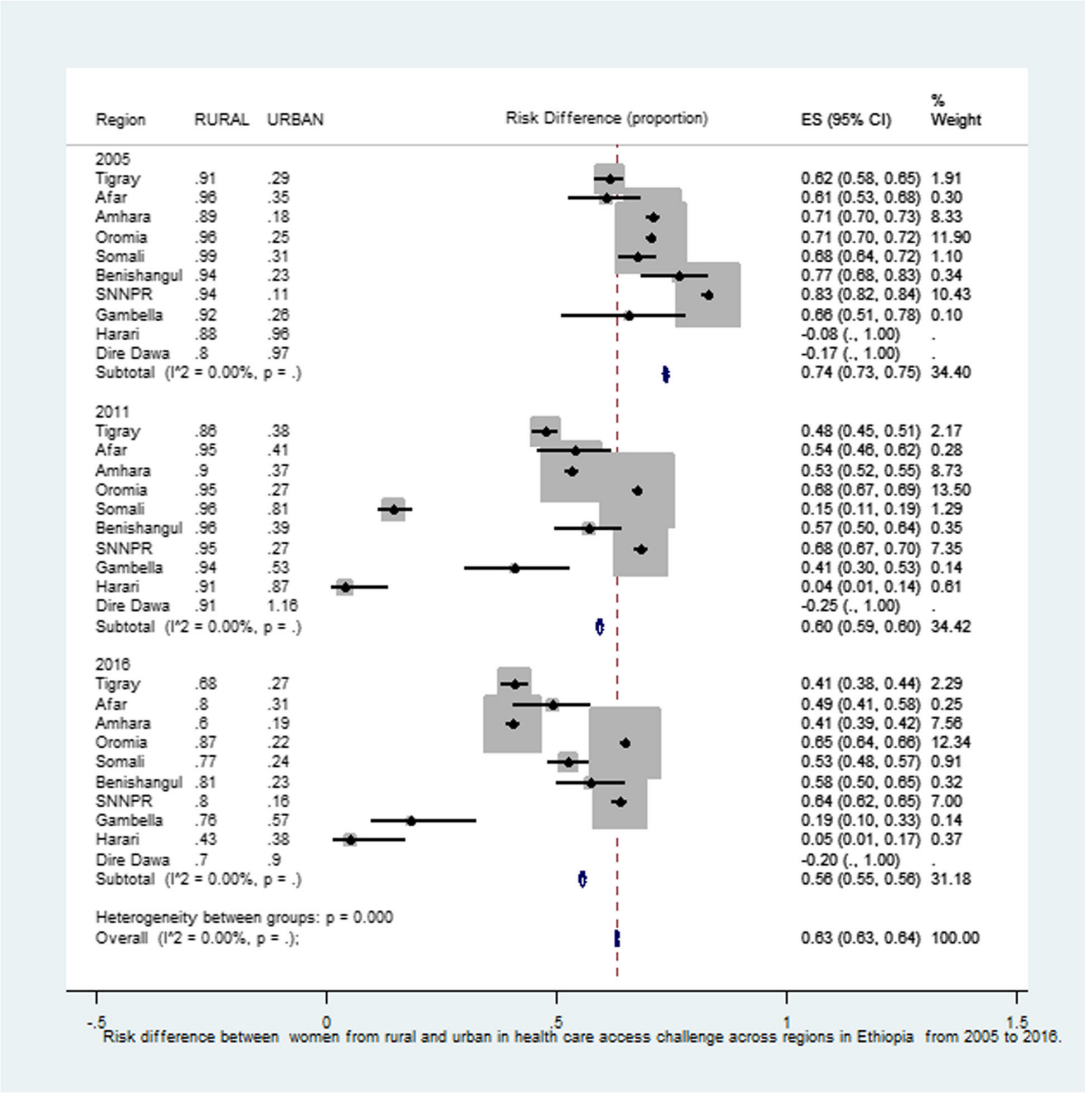
Forest plot of risk difference between women from urban and rural area in health care access challenge across regions in Ethiopia from 2005 to 2016

**Fig. 4:**
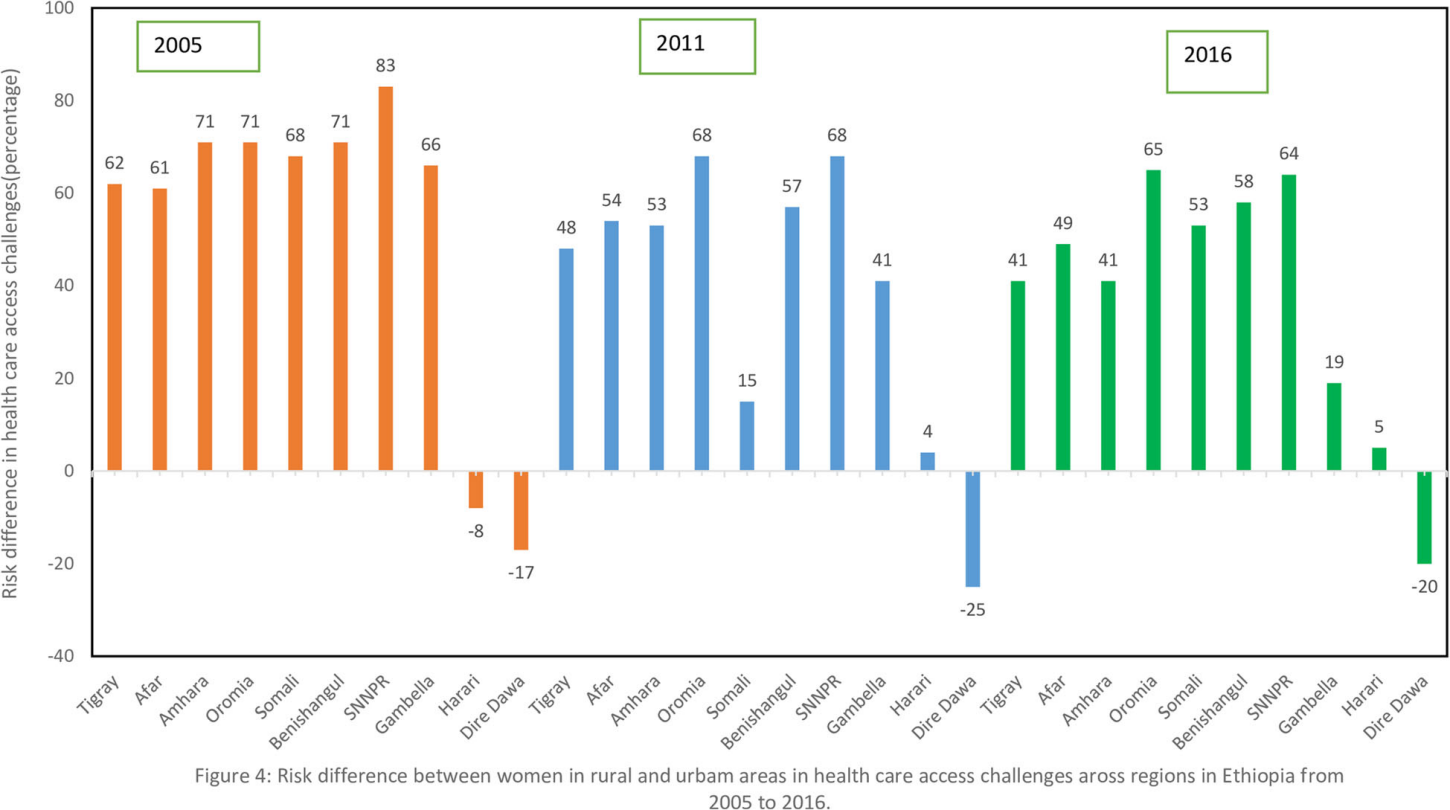
Risk difference between women in rural and urban area across regions in Ethiopia over time

Figure 3 and Fig. 4 show that the risk difference in women health care access challenges across regions over time (2005–2016) in Ethiopia. the risk difference in women health care access challenges were significantly varied across regions in Ethiopia across the three EDHS surveys (EDHS 2005, 2011 and 2016). In EDHS 2005, overall there was significant risk difference in health care access challenges across residence *(RD = 0.74, 95% CI: 0.73, 0.75).* The highest significant urban-rural health care challenge inequality were observed in SNNP region which was *(RD = 0.82, 95%: 0.82, 0.84)* followed by Benishangul-Gumuz (RD *= 0.77, 95% CI: 0.68, 0.83).* In EDHS 2011, overall there was significant urban-rural difference in health care access challenges in Ethiopia *(RD = 0.60, 95% CI: 0.59, 0.60)*. The highest risk difference was observed SNNPR *(RD = 0.68, 95% CI: 0.67, 0.70)* and Oromia region *(RD = 0.68, 95% CI: 0.67, 0.69)* while the lowest risk difference was observed in Harari region *(RD = 0.04, 95% CI: 0.01, 0.14).* In EDHS 2016, there was singnificant risk difference in women health care challenge between urban and rural areas across regions in Ethiopia *(RD = 0.63, 95% CI: 0.63, 0.64).* The highest residential inequality in health care access challenges was observed in oromia regions (*RD = 0.65, 95% CI: 0.64, 0.66)* while the lowest risk difference in Harari region *(RD = 0.05, 95% CI: 0.01, 0.17).*

### Spatial distribution of health care access challenges

**Fig. 5:**
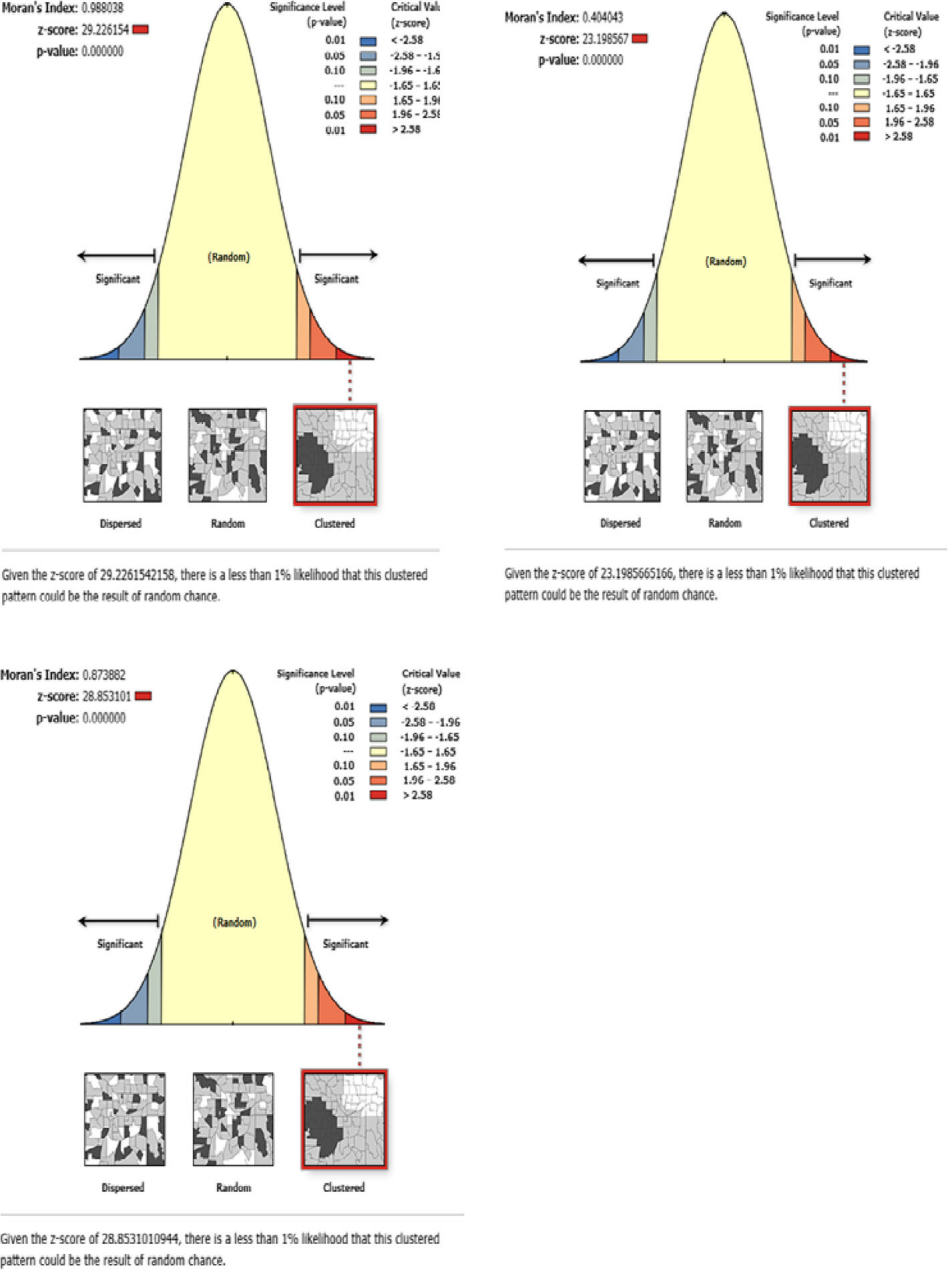
The global spatial autocorrelation of health care access challenges in Ethiopia 2005, 2011 and 2016

**Fig. 6:**
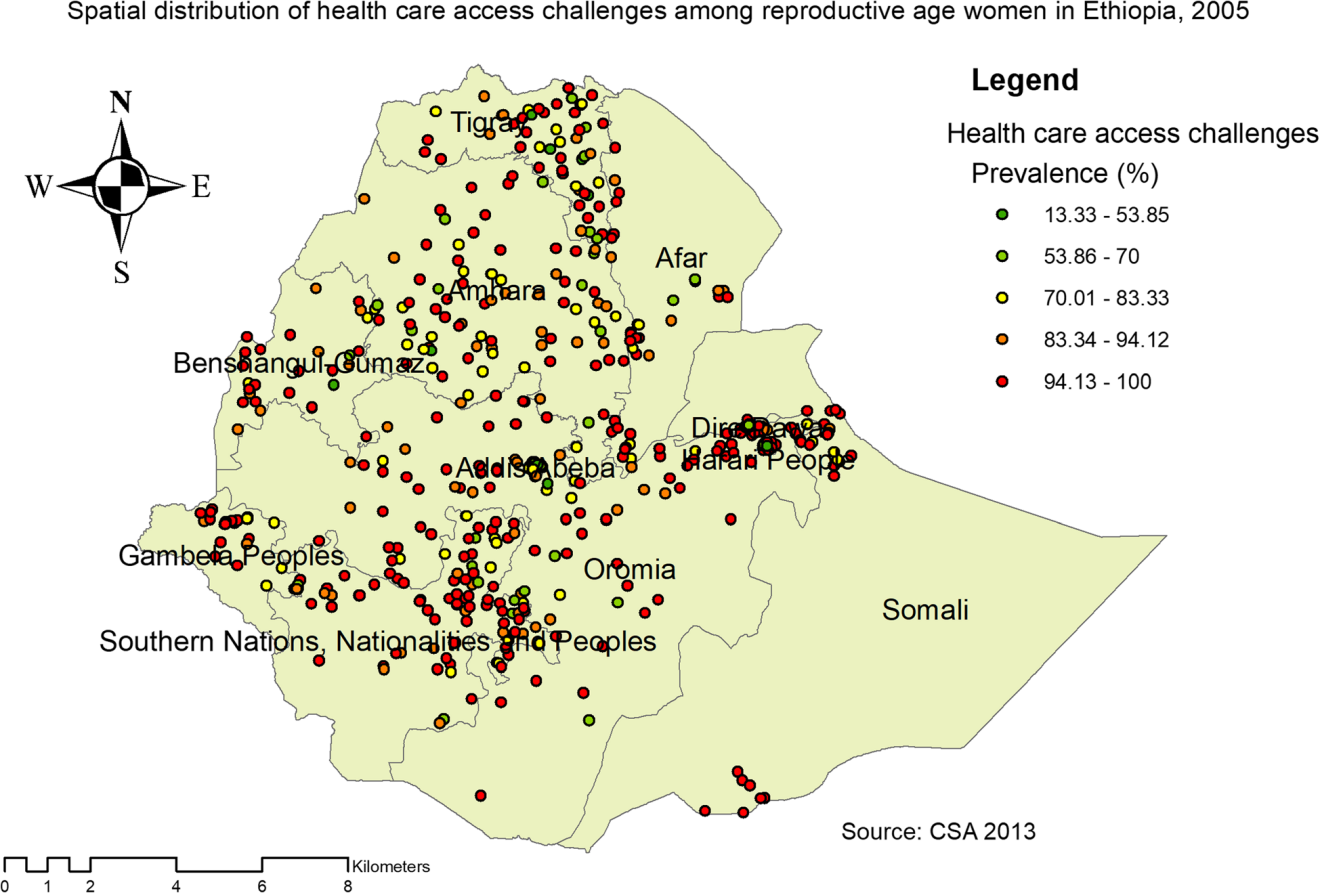
The spatial distribution of health care access challenges among reproductive-age women in Ethiopia, 2005 (Source: CSA 2013, using Arc-GIS version 10.6 and SaTScan version 9.6 statistical software)

**Fig. 7:**
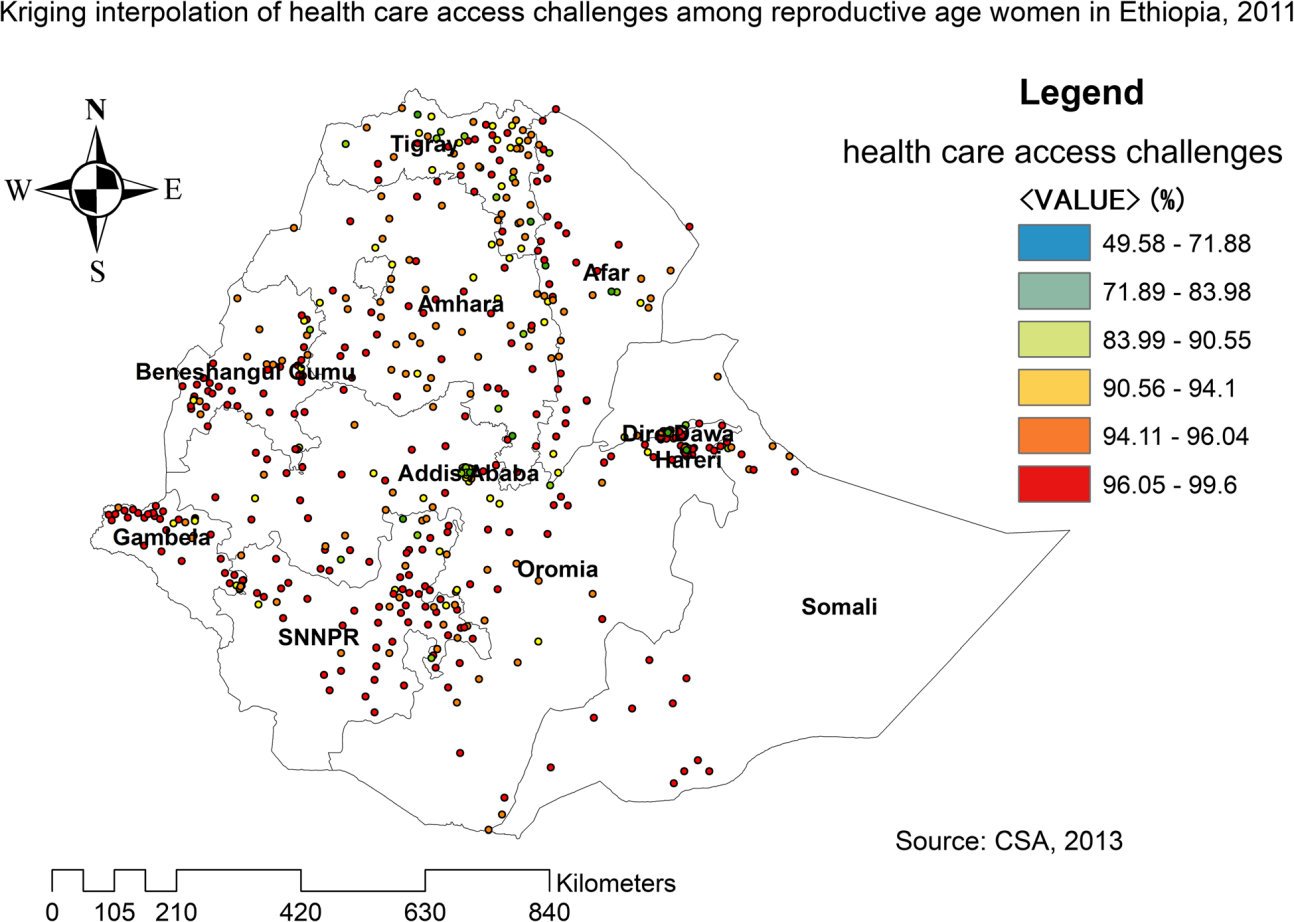
The spatial distribution of health care access problem challenges among reproductive-age women in Ethiopia, 2011 (Source: CSA 2013, using Arc-GIS version 10.6 and SaTScan version 9.6 statistical software)

**Fig. 8:**
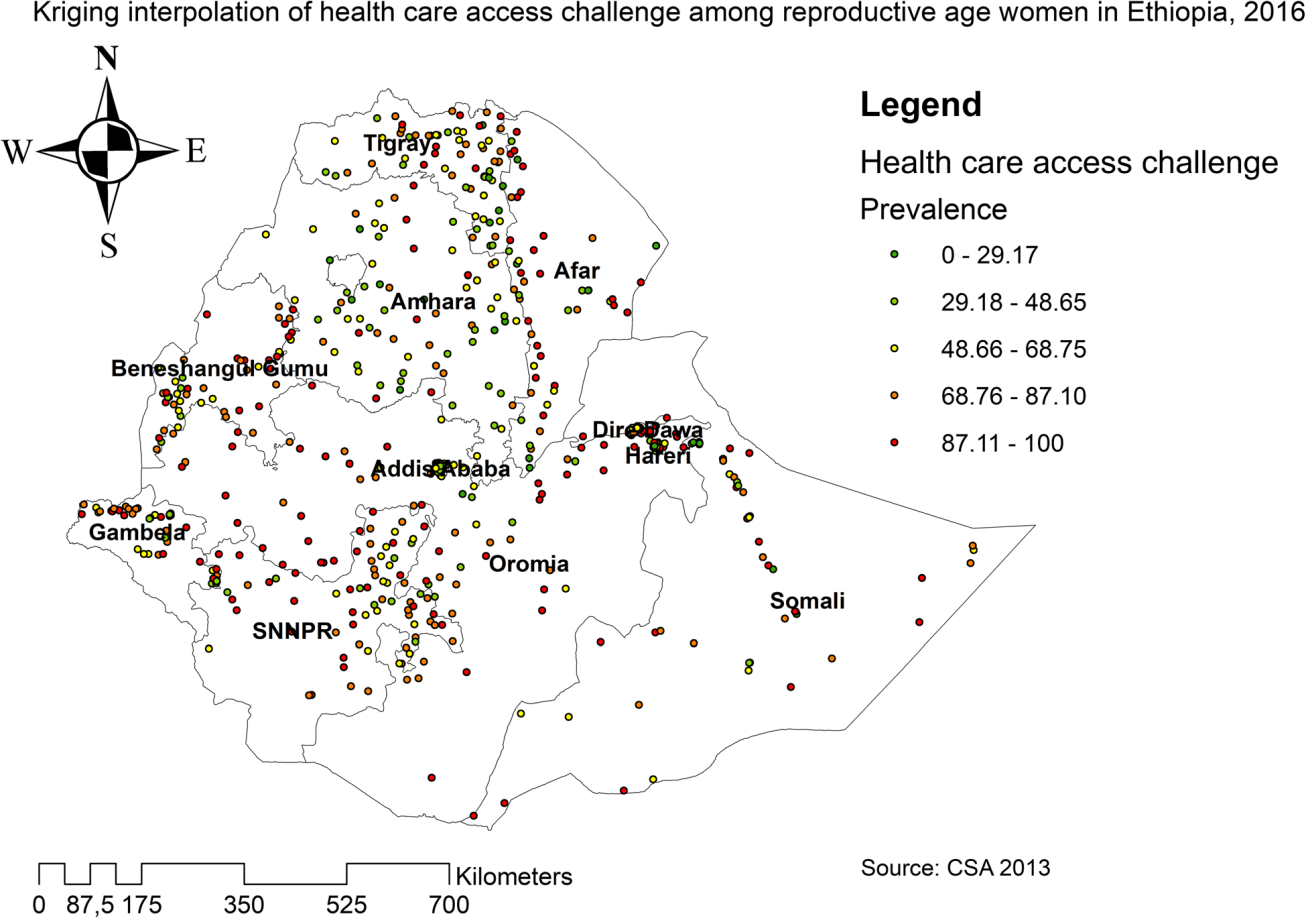
The spatial distribution of health care access challenges among reproductive-age women in Ethiopia, 2016 (Source: CSA 2013, using Arc-GIS version 10.6 and SaTScan version 9.6 statistical software)

The spatial distribution of health care challenges showed significant spatial variation across the country over time (Fig. 5). The highest prevalence of health care access challenges was identified in Somali, Harari, Benishangul Gumuz, east SNNPRs, and Afar regions consistently over time (Figs. 6, 7, and 8).

**Fig. 9:**
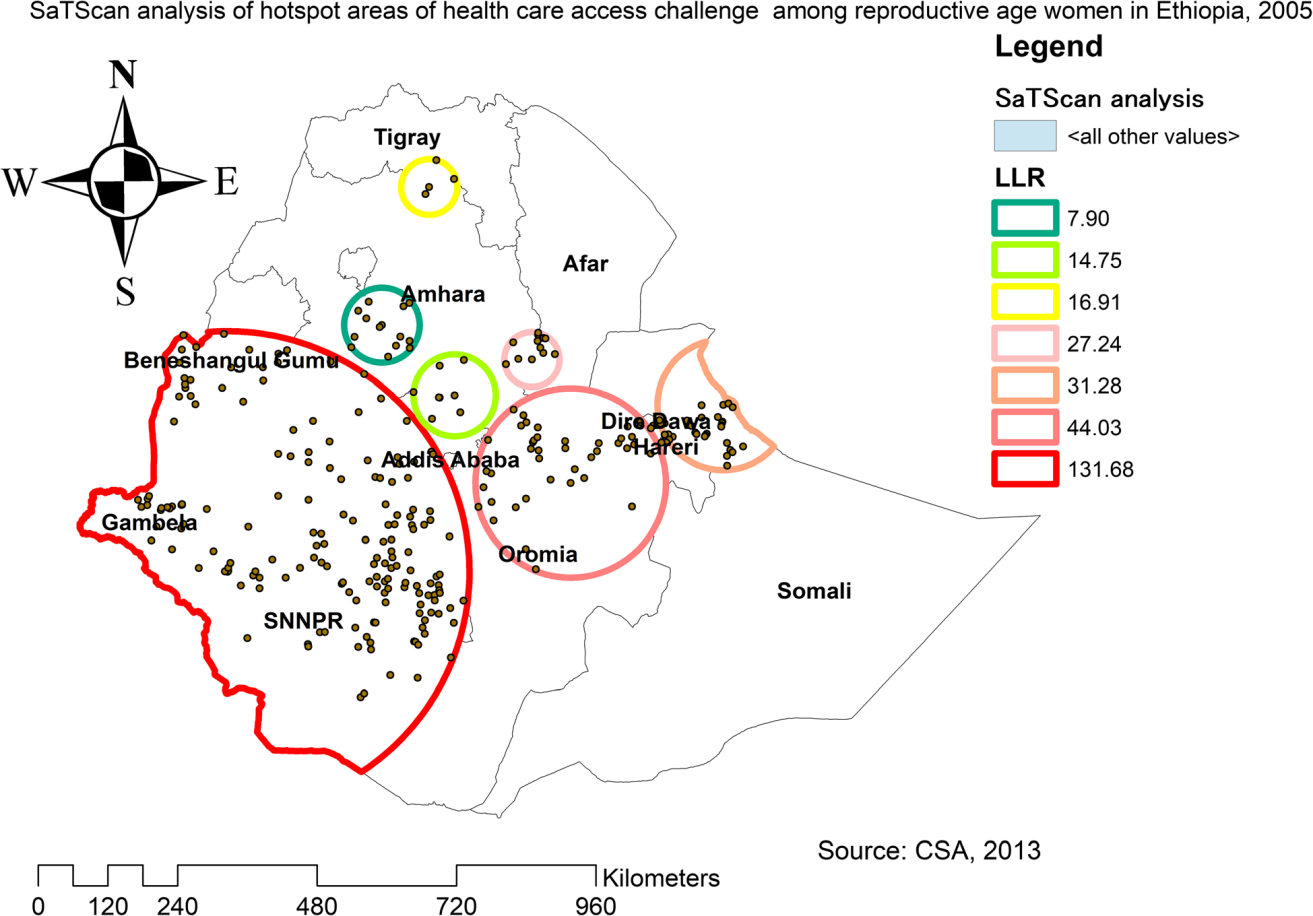
The SaTScan analysis of hotspot areas of women health care access challenges among reproductive-age women in Ethiopia, 2005 (Source: CSA 2013, using Arc-GIS version 10.6 and SaTScan version 9.6 statistical software)

In EDHS 2005, the spatial scan statistics identified a total of 286 primary and secondary clusters of health care access challenges. Of these, 173 clusters were most likely (primary cluster), the spatial window was located in Gambela, SNNPR, south Benishangul and southwest Oromia regions centered at 7.222698 N, 35.310296 E with 415.38-km radius, a Relative Risk (RR) of 1.12 and Log-Likelihood (LLR) of 131.7, at *p*-value < 0.01 (Table 5). It showed that women within the spatial window had 1.12 times higher likelihood of health care access challenges as compared to women outside the spatial window. Whereas the secondary clusters were located in central Oromia, Dire Dawa, Harari, northeast Somali, and central Amhara regions (Fig. 9).

**Table 5:** SaTScan analysis result of health care access challenges among reproductive age women in Ethiopia, 2005

Cluster	Enumeration area (cluster)identified	Coordinate/radius	Population	Case	RR	LLR	*p*-value
1 (173)	21, 531, 282, 445, 315, 171, 459, 60, 421, 299, 9, 355, 507, 474, 482, 184, 526, 329, 167, 319, 104, 266, 163, 333, 134, 485, 312, 222,81, 533, 325, 406, 151, 372, 487, 336, 395, 387, 465, 328, 409, 73,503, 219, 119, 153, 400, 284, 236, 130, 436, 168, 67, 394, 269, 534,276, 94, 419, 201, 512, 35, 122, 365, 504, 193, 224, 292, 514, 53, 249, 30, 13, 65, 464, 105, 46, 519, 68, 34, 517, 12, 537, 317, 45,235, 279, 467, 283, 273, 202, 107, 480, 209, 131, 535, 432, 126, 434,373, 255, 497, 118, 210, 305, 340, 339, 48, 5, 420, 144, 252, 323,499, 170, 343, 172, 518, 359, 342, 80, 254, 40, 251, 189, 234, 199,7, 77, 280, 380, 230, 286, 444, 83, 441, 61, 239, 258, 229, 274, 8455, 389, 245, 49, 363, 539, 28, 206, 520, 191, 324, 90, 376, 44,352, 489, 457, 113, 347, 213, 212, 215, 296, 69, 361, 353, 112, 146, 244, 435	(7.222698 N, 35.310296 E) / 415.38 km	4479	4114	1.12	131.7	0.000001
2 (41)	33, 100, 486, 290, 247, 6, 443, 242, 344, 265, 334, 174, 442, 498,492, 54, 14, 460, 137, 407, 78, 297, 314, 383, 88, 138, 522, 31, 233,165, 16, 397, 232, 392, 298, 379, 198, 309, 142, 291, 32	(8.617232 N, 40.661954 E) / 163.66 km	941	889	1.12	44.0	0.00001
3 (31)	52, 538, 176, 58, 127, 50, 241, 108, 374, 466, 221, 43, 494, 197,508, 313, 183, 404, 438, 4, 253, 106, 25, 41, 208, 375, 453, 169,524, 141, 272	(9.813151 N, 43.060046 E) / 112.86 km	653	618	1.12	31.3	0.0001
4 (12)	377, 55, 56, 410, 513, 148, 481, 385, 101, 82, 250, 57	(10.541555 N, 40.061798 E) / 47.44 km	309	301	1.15	27.2	0.001
5 (4)	448, 423, 205, 150	(13.227055 N, 38.440911 E) / 48.24 km	104	104	1.18	16.9	0.002
6 (8)	458, 98, 214, 218, 270, 264, 181, 211	(9.985926 N, 38.846195 E) / 70.76 km	203	196	1.14	14.8	0.003
7 (14)	304, 354, 231, 461, 182, 115, 97, 351, 1, 338, 415, 479, 99, 322	(11.075622 N, 37.699792 E) / 64.86 km	367	337	1.08	7.90	0.107
8 (3)	133, 509, 440	(9.254234 N, 42.126181 E) / 4.57 km	72	69	1.13	4.42	0.98

**Fig. 10:**
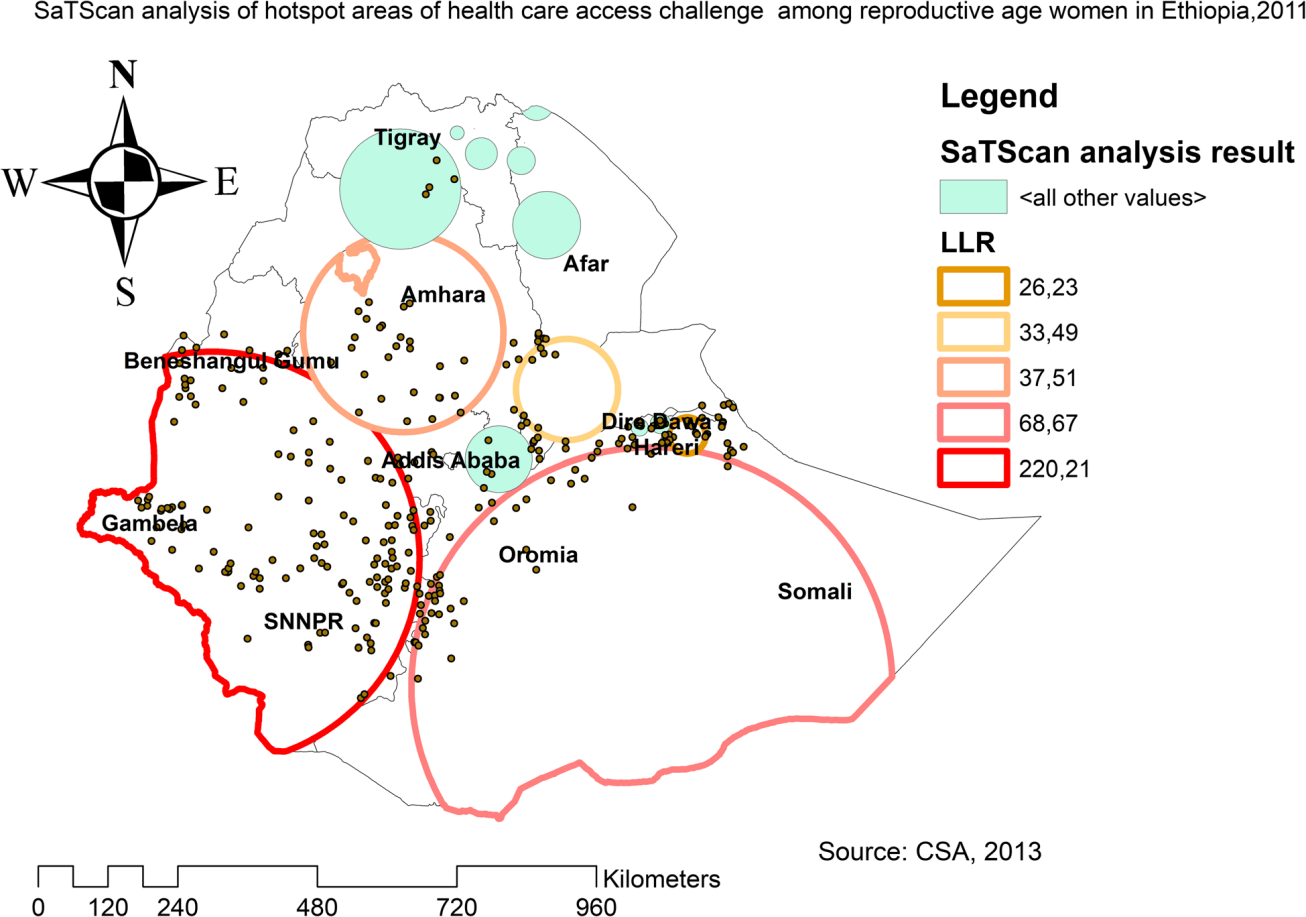
The SaTScan analysis of hotspot areas of women health care access challenges among reproductive-age women in Ethiopia, 2011 (Source: CSA 2013, using Arc-GIS version 10.6 and SaTScan version 9.6 statistical software)

**Fig. 11:**
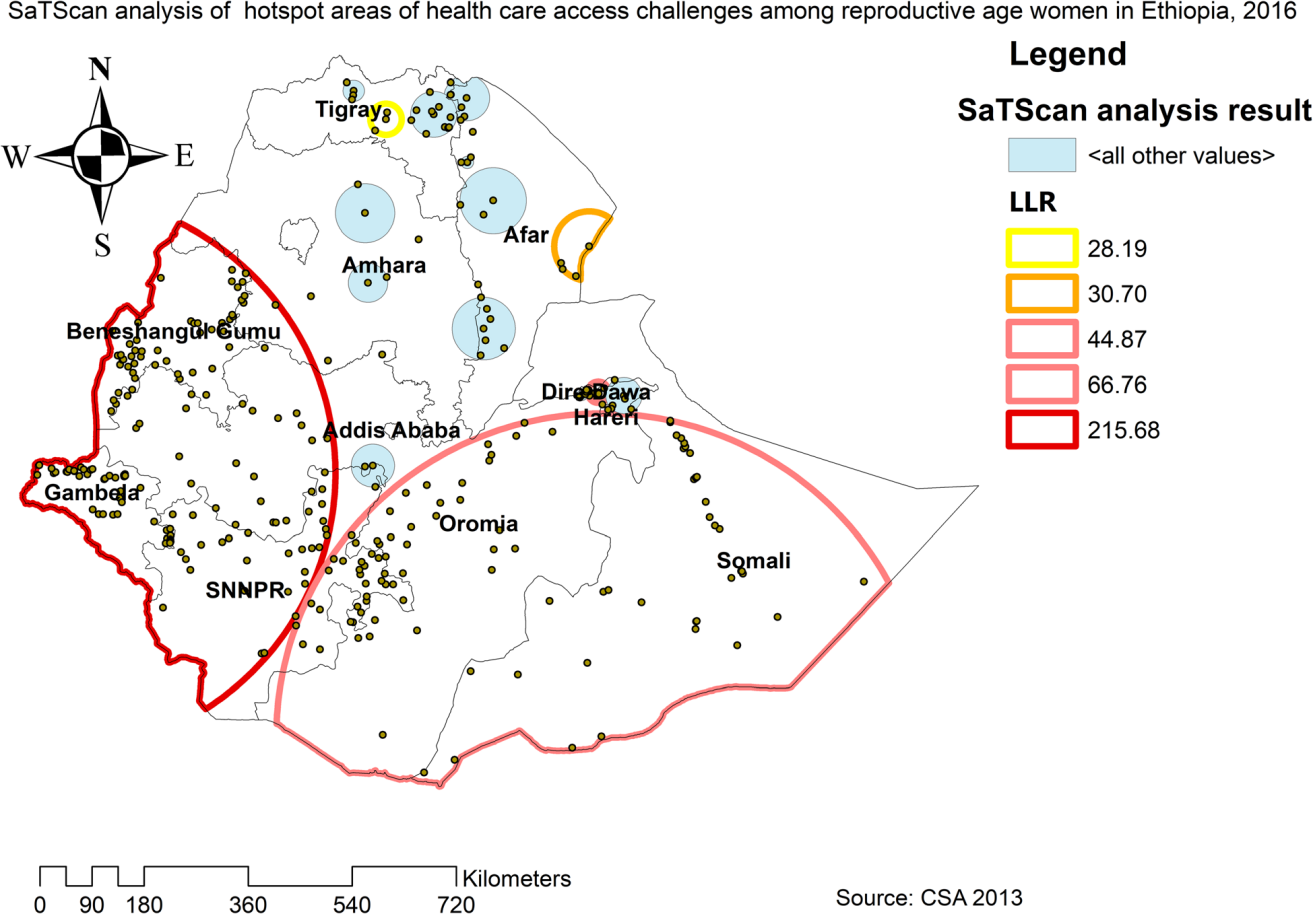
The SaTScan analysis of hotspot areas of women health care access challenges among reproductive-age women in Ethiopia, 2016 (Source: CSA 2013, using Arc-GIS version 10.6 and SaTScan version 9.6 statistical software)

In EDHS 2011, the spatial scan statistics identified a total of 286 primary and secondary clusters of health care access challenges. Of these, 146 clusters were most likely clusters, which was located in south Benishangul, Gambela, SNNPR, and southwest Oromia regions, centered at 7.437690 N, 35.059032 E with 356.58-km radius, a Relative Risk (RR) 1.16, and Log-Likelihood Ratio (LRR) of 220.2, at *p*-value< 0.01 (Table 6). It showed that women within the spatial window had 1.16 times higher likelihood of health care access challenges as compared to women outside the spatial window. Whereas the secondary clusters were located in the Somali, Harari, Afar, and central Amhara regions (Fig. 10). In EDHS 2016, the SaTScan statistics identified a total of 280 primary and secondary clusters of these, of these 153 were most likely clusters which were located in SNNPR, Gambella and Benishangul regions centered at 8.268721 N, 33.486779 E with 485.32 km radius, RR of 1.31 and LLR of 215.9, at *p*-value< 0.01 (Table 7). It showed that women within the spatial window had a 1.31 times higher likelihood of health care access challenges as compared to women outside the spatial window (Fig. 11). Overall the SaTScan analysis revealed that Gambella, SNNPR, and Benishangul regions were persistently at higher risk of health care access challenges across the three surveys.

**Table 6:** SaTScan analysis result of health care access challenges among reproductive age women in Ethiopia, 2011

Cluster	Enumeration area (cluster)identified	Coordinate/radius	Population	Case	RR	LLR	*p*-value
1 (146)	30, 107, 545, 354, 160, 511, 368, 223, 639, 559, 631, 327, 125, 389,267, 530, 457, 315, 396, 375, 404, 207, 409, 586, 339, 206, 116, 364,383, 648, 492, 487, 624, 243, 434, 395, 422, 633, 477, 588, 185, 536,196, 520, 25, 547, 447, 642, 259, 277, 598, 475, 158, 490, 454, 161,390, 562, 261, 505, 470, 63, 264, 401, 405, 580, 135, 553, 324, 283,105, 610, 619, 607, 301, 578, 468, 239, 113, 552, 427, 341, 342, 294,622, 273, 317, 349, 61, 252, 449, 242, 130, 432, 132, 443, 411, 412,641, 360, 563, 357, 435, 254, 332, 232, 378, 12, 625, 408, 248, 466,157, 100, 81, 202, 282, 112, 262, 14, 97, 337, 257, 6, 284, 16, 329, 276, 40, 303, 472, 142, 229, 27, 391, 526, 47, 8, 513, 166, 605, 438, 149, 143, 204, 382	(7.437690 N, 35.059032 E) / 356.58 km	3910	3660	1.16	220.2	0.000001
2 (49)	250, 627, 240, 416, 48, 295, 381, 346, 266, 187, 279, 5, 425, 350, 377, 93, 75, 233, 176, 145, 17, 646, 471, 321, 518, 583, 234, 541,56, 291, 351, 198, 307, 37, 402, 297, 558, 42, 344, 7, 442, 310, 320,570, 55, 147, 172, 358, 121	(5.462248 N, 41.898267 E) / 412.18 km	1301	1223	1.14	68.7	0.00001
3 (46)	557, 426, 89, 484, 388, 20, 43, 18, 356, 181, 90, 407, 635, 151, 35,453, 123, 439, 288, 473, 398, 119, 218, 104, 551, 621, 628, 585, 29,171, 369, 255, 554, 271, 227, 334, 615, 38, 188, 300, 437, 219, 555,372, 1, 115	(10.963489 N, 38.032582 E) / 171.95 km	1336	1222	1.10	37.5	0.0001
4 (8)	26, 85, 421, 509, 423, 577, 80, 514	(10.078026 N, 40.604658 E) / 87.64 km	213	212	1.19	33.5	0.0001
5 (8)	120, 359, 532, 313, 503, 28, 594, 340	(9.349054 N, 42.484599 E) / 31.36 km	190	188	1.19	26.2	0.001
6 (7)	191, 433, 66, 581, 65, 133, 231	(12.635948 N, 40.297925 E) / 58.30 km	167	166	1.19	25.4	0.003
7 (5)	117, 53, 228, 87, 124	(8.998897 N, 39.531366 E) / 57.46 km	146	143	1.17	16.4	0.005
8 (17)	174, 347, 504, 462, 333, 460, 637, 64, 403, 592, 195, 77, 154, 217, 245, 180, 249	(13.197632 N, 37.977752 E) / 104.03 km	492	450	1.10	13.2	0.0011

**Table 7:** SaTScan analysis result of health care access challenges among reproductive age women in Ethiopia, 2016

Cluster	Enumeration area (cluster)identified	Coordinate/radius	Population	Case	RR	LLR	*p*-value
1 (153)	435, 536, 309, 370, 507, 592, 266, 618, 260, 104, 233, 69, 426, 603, 346, 315, 567, 343, 13, 105, 417, 106, 284, 265, 221, 549, 231, 593, 291, 47, 469, 63, 114, 270, 219, 446, 643, 448, 193, 248, 275, 175, 395, 462, 326, 374, 17, 119, 558, 46, 165, 416, 554, 6, 526, 197,299, 317, 203, 243, 508, 459, 465, 552, 168, 371, 581, 433, 555, 595,177, 563, 304, 407, 335, 437, 409, 209, 285, 324, 621, 325, 349, 124, 65, 569, 477, 586, 376, 70, 88, 457, 337, 207, 411, 154, 489, 320, 62, 161, 244, 76, 35, 364, 137, 294, 432, 338, 183, 184, 150, 256,470, 486, 36, 447, 280, 559, 227, 246, 533, 494, 306, 399, 234, 498,615, 502, 142, 113, 118, 515, 548, 386, 406, 174, 126, 541, 141, 577,262, 415, 331, 434, 565, 23, 466, 87, 41, 602, 109, 360, 450	(8.268721 N, 33.486779 E) / 485.32 km	3551	2774	1.31	215.9	0.000001
2 (94)	520, 208, 556, 394, 278, 164, 187, 480, 377, 318, 7, 358, 85, 138,82, 289, 492, 286, 146, 422, 92, 543, 472, 490, 601, 452, 171, 198,34, 95, 398, 316, 497, 518, 405, 21, 468, 313, 232, 600, 576, 445,182, 26, 521, 574, 588, 562, 32, 123, 553, 458, 634, 365, 619, 213,12, 319, 589, 215, 216, 308, 391, 408, 50, 148, 214, 578, 529, 251,573, 245, 77, 239, 524, 503, 522, 116, 372, 22, 342, 347, 438, 609, 476, 122, 505, 20, 420, 162, 568, 412, 277, 86	(4.180558 N, 42.052871 E) / 561.30 km	2262	1683	1.20	66.8	0.00001
3 (10)	281, 523, 242, 311, 642, 166, 202, 352, 613, 514	(9.561432 N, 42.037215 E) / 18.18 km	243	219	1.42	44.9	0.0001
4 (4)	4, 632, 75, 596	(11.845228 N, 41.915793 E) / 60.32 km	94	91	1.52	30.7	0.0001
5 (3)	156, 636, 551	(13.848685 N, 38.688109 E) / 27.17 km	88	85	1.52	28.2	0.001
6 (6)	1, 566, 622, 186, 307, 436	(9.505470 N, 42.438628 E) / 31.16 km	118	105	1.40	19.5	0.003
7 (2)	199, 628	(12.376936 N, 38.357984 E) / 51.17 km	43	43	1.57	19.3	0.001
8 (3)	130, 511, 172	(13.169308 N, 39.987117 E) / 10.69 km	71	67	1.48	18.8	0.01
9 (5)	594, 557, 441, 30, 380	(9.520494 N, 41.780549 E) / 9.19 km	118	104	1.38	18.0	0.02

### Kriging interpolation of health care access challenges

**Fig. 12:**
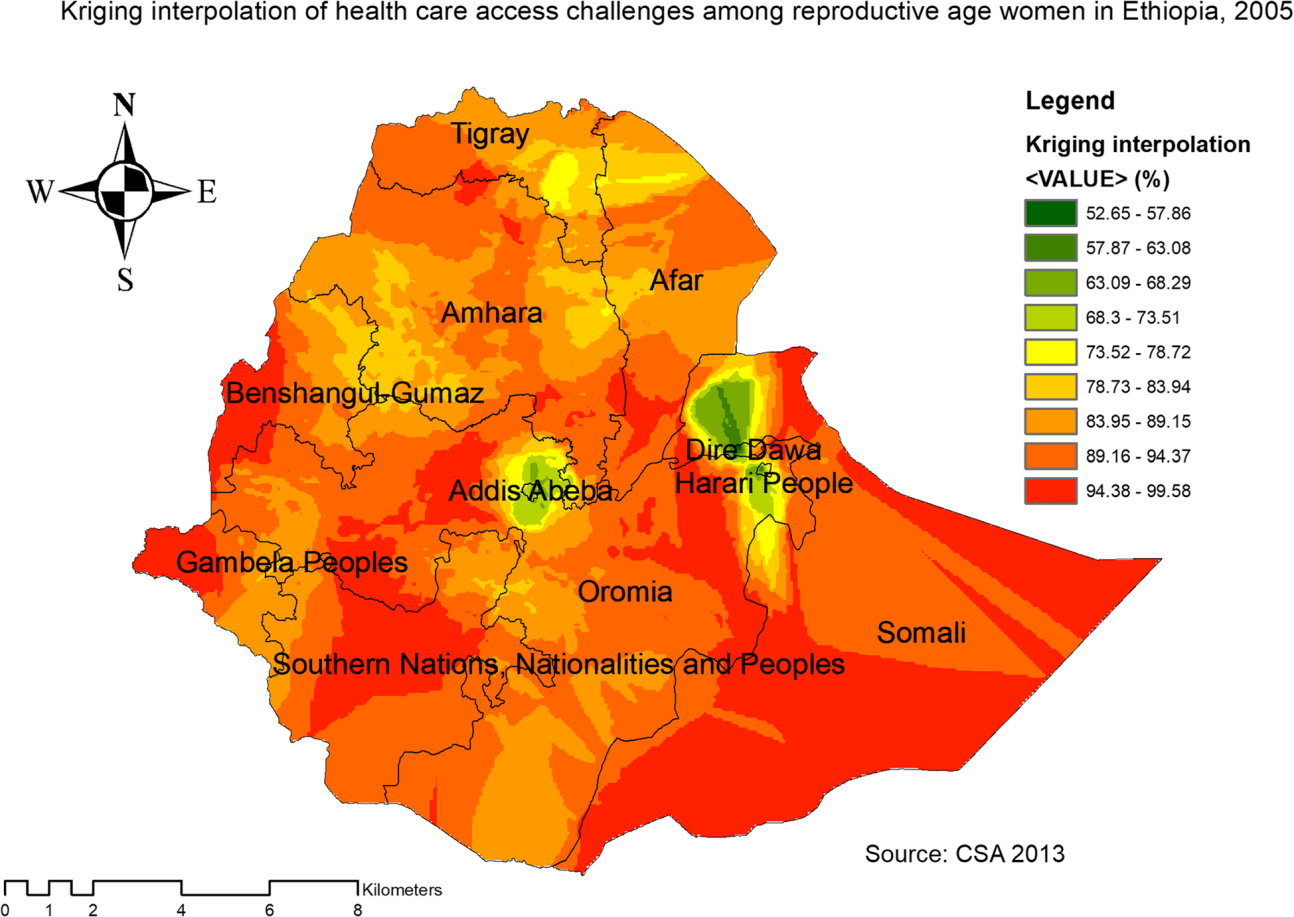
The Kriging interpolation of health care access challenges among reproductive-age women in Ethiopia, 2005 (Source: CSA 2013, using Arc-GIS version 10.6 and SaTScan version 9.6 statistical software)

Based on EDHS 2005, Kriging interpolation predict that the highest health care access challenges were detected in the southern and eastern part of Somali, east SNNPR, west Benishangul, and Gambella regions whereas, predicted relatively low health care access challenge located in the Addis Ababa, south Oromia and Dire Dawa (Fig. 12).

**Fig. 13:**
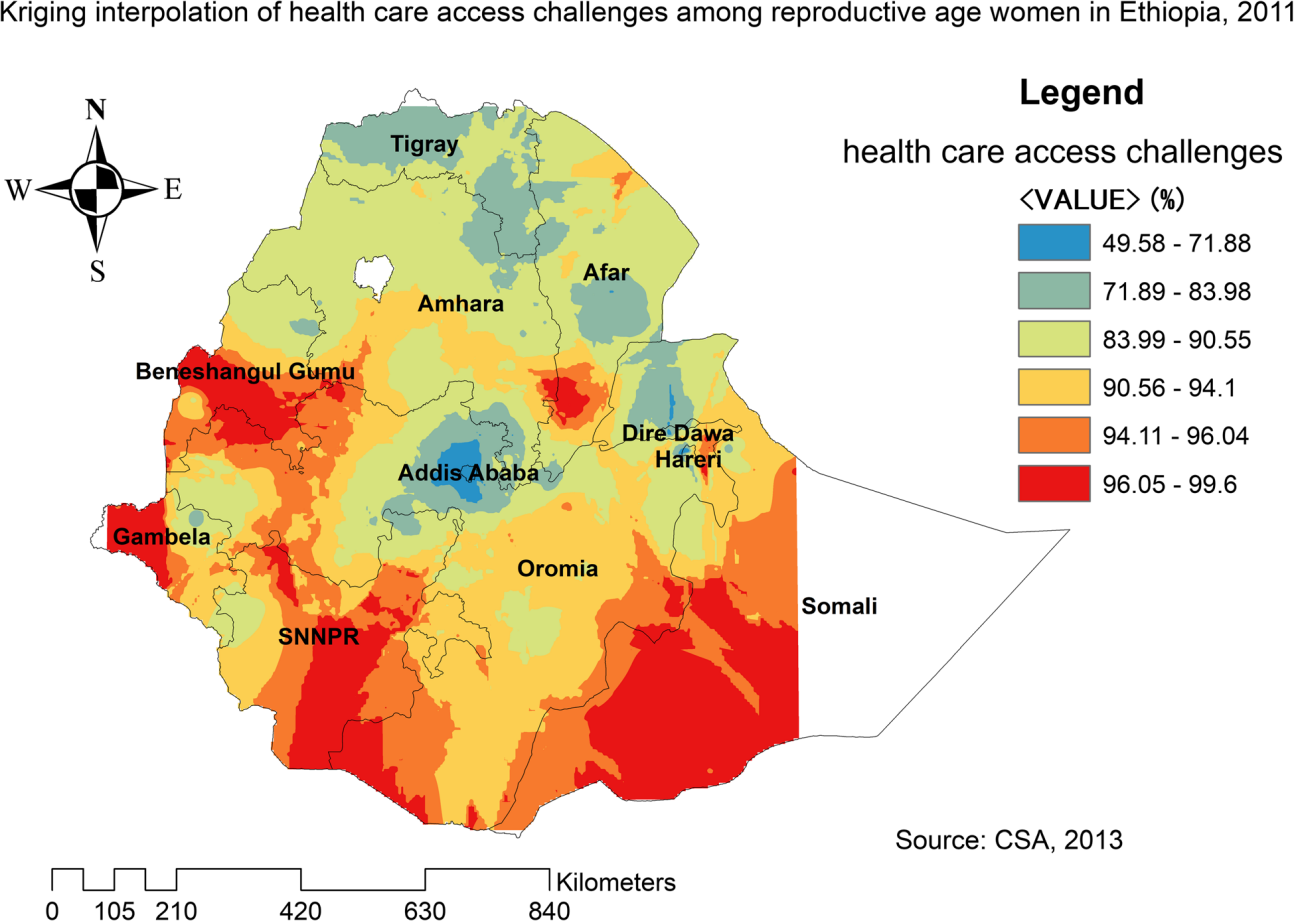
The Kriging interpolation of health care access challenges among reproductive-age women in Ethiopia, 2011 (Source: CSA 2013, using Arc-GIS version 10.6 and SaTScan version 9.6 statistical software)

**Fig. 14:**
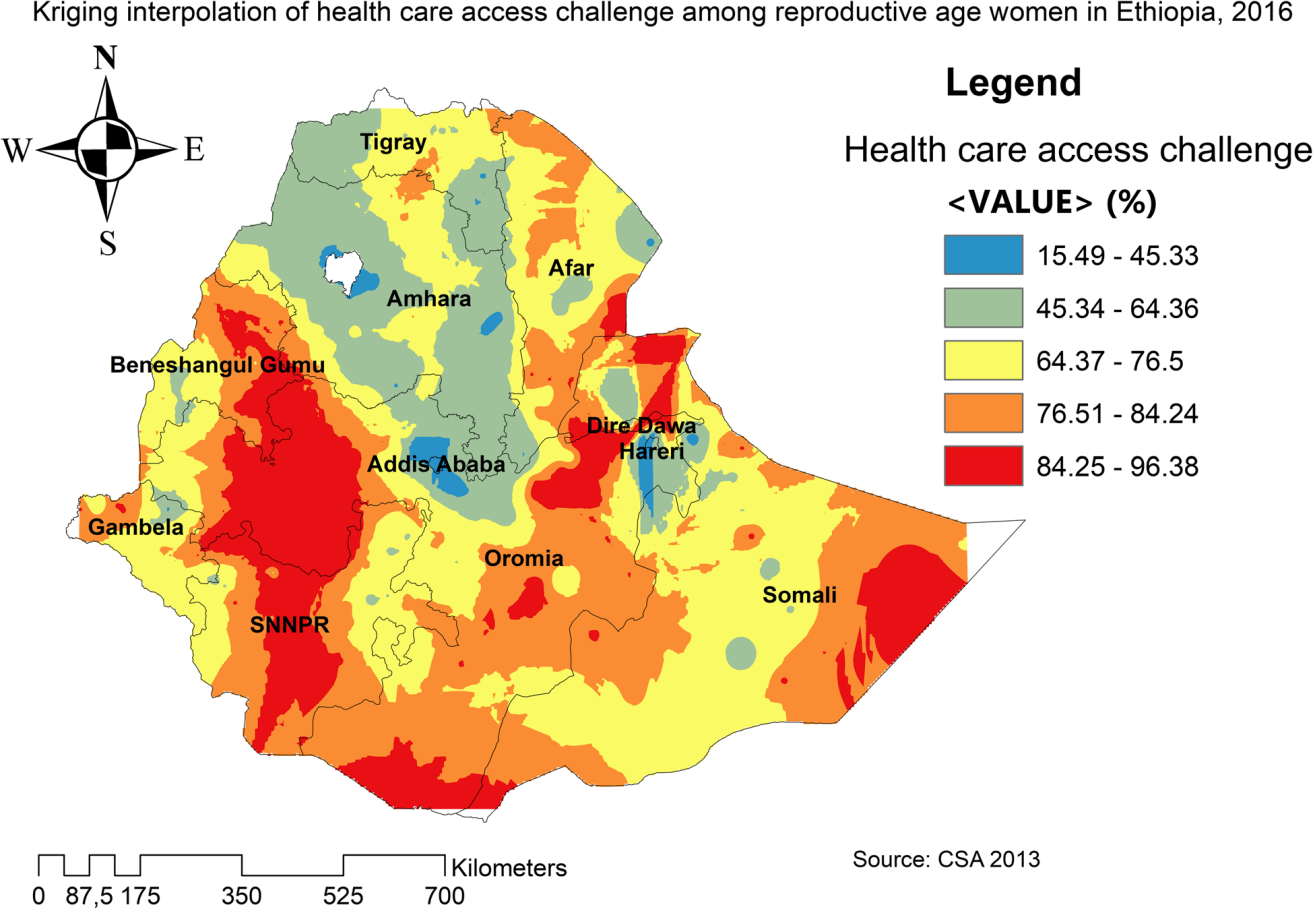
The Kriging interpolation of health care access challenges among reproductive-age women in Ethiopia, 2016 (Source: CSA 2013, using Arc-GIS version 10.6 and SaTScan version 9.6 statistical software)

In 2011, Kriging interpolation revealed that the highest predicted prevalence of health care access challenges was found in Benishangul, west Gambella, SNNPR, south Oromia and Somali regions. In contrast, predicted low health care access challenges were detected in Tigray, Afar, Amhara, Addis Ababa, Harari, and Dire Dawa (Fig. 13). From EDHS 2016 data, Kriging interpolation predicted that east Somali, southeast, and west Oromia, central SNNPR, and east Benishangul contained the highest health care access problem while Tigray, Addis Ababa, and Amhara regions contained relatively low health care access problem (Fig. 14).

## Discussion

Over the last 15 years, Ethiopia has achieved different socioeconomic development and health system improvements reflected by a reduction of maternal mortality, increased per-capita, and life expectancy of citizens. Besides, remarkable improvements were also shown with increased health facility coverage in the population with the construction of different health facilities and the deployment of health professionals and resources [6, 41].

Trend analysis in this study showed that over 10 years the magnitude of health access perceived barriers/ challenges among reproductive-age women has decreased from 96% in 2005 to 70% 2016, according to Ethiopian demographic and health survey data [6, 20]. There was significant risk difference in health care access challenges between urban and rural areas across regions over the three surveys. About 14.8% of the overall reduction of health care access perceived challenges was attributed to the change in the composition of the respondents and the remaining 85.2% of the overall decrease in health care perceived barriers were due to the change in the effects of explanatory variables (coefficients). Population structure changes such as increased literacy level and improvement of socio-demographic and economic characteristics contributed to the reduction of health care access problems among reproductive-age women in Ethiopia. In addition, government commitment to the realization of the millennium development goal (MDG) through the provision of maternal health care services free of charge may also be contributed to the reduction of health care access barriers. This finding was supported by previous studies [42–44].

Socio-demographic characteristics like husband and woman level of education, home delivery, residence, female household head, and had previous ANC follow up were factors contributed to the overall changes health access perceived barriers among reproductive-age women in the last 10 years. This finding was consistent with previous studies in Ethiopia and Tanzania [36, 43]. Also, increased health-seeking behavior and through the implementation of health extension programs which increased accessibility and availability of health services at the grassroots level. Particularly, female household head and women age 25–34 years old associated with decreased perceived barriers of health care access, this could be since female autonomy could increase health-seeking behavior [45].

Findings from the spatial analysis showed that health access problems distributions were not random. The highest health care access problem was spatially clustered in Somali, Harari, Benishangul Gumuz, east SNNPRs, and Afar regions consistently over time as depicted in Fig. 4. This finding was consist of previous studies in Ethiopia [18, 43, 46]. This could be because the above-mentioned areas/regions are less developed and the majority of habitats are pastoralists with no permanent residence to establish health facilities and provide services. Besides, the socio-demographic characteristics of society might affect the health-seeking behavior of women like cultural barriers.

In addition to this spatial interpolations also the highest magnitude of health care access problems was detected in Benishangul, west Gambella, SNNPR, south Oromia, and Somali regions of Ethiopia. This finding was consistent with previous studies [18]. These regions are who most of the populations are pastoralists and there are documented security issues that might affect health access of the population.

This study had several strengths. Firstly, the study was based on nationally representative large datasets, and thus, it has adequate statistical power. Secondly, the estimates of the study were done after the data were weighted for the probability sampling and non-response, to make it representativeness at national and regional levels: therefore, it can be generalized to all reproductive-age women in the study setting. Thirdly, multivariate decomposition analysis was applied to understand the sources of changes in health care access problems over time. Finally, the use of GIS and SaTScan statistical tests helped to detect similar and statistically significant hotspot areas of health care access problems across the surveys and to design effective public health programs.

Limitations, the outcome variables were not collected in EDHS 2000. The other limitation was, the SaTScan detect only circular clusters, and irregularly shaped clusters were not detected. Furthermore, the EDHS survey did not incorporate community-level variables like community norm, culture, and beliefs and medical factors rather it relied on mothers or caregivers report and might have the possibility of social desirability and recall bias through CSA claim that strong effort was made to minimize it mainly through extensive training of data collectors, recruiting experienced data collectors and supervisors this might underestimate our finding.

## Conclusion

Perceived health care access challenges have shown a remarkable decrease over time but there was variation in barriers to health care access across Ethiopia. Media exposure improved mothers’ health care access in Ethiopia. Public health programs targeting rural, uneducated, unemployed, and women whose husband had no education would be helpful to alleviate health care access problems in Ethiopia. Socio-demographic characteristics like partner and women’s level of education, home delivery, and ANC utilization were factors that contributed to the observed changes over the last decades. Besides, health access problems were not randomly distributed and south and eastern Ethiopia were regions of high health access problems. This suggests that further public health interventions are important for further reduction of health care access barriers through the uplifting socio-demographic and economic status of the population.

## Data Availability

Data we used for this study is publicly available in the MEASURE DHS program and you can access it from www.measuredhs.com after explaining the objectives of the study. Then after receive the authorization letter, the data is accessible and freely downloaded.

## Abbreviations

CI: Confidence interval
DHS: Demographic health survey
EAs: Enumeration areas
EDHS: Ethiopian demographic and health survey
LLR: Log likelihood ratio
LR: Likelihood ratio
RR: Relative risk
SNNP: Southern nations and nationalities of people
SSA: Sub-Saharan Africa
